# Role of surgeon seniority in predicting surgically induced astigmatism after phacoemulsification surgery: a machine learning study

**DOI:** 10.1186/s12886-026-05203-y

**Published:** 2026-08-06

**Authors:** Denizcan Özizmirliler, Ceren Durmaz Engin, Özlem Özkan, Furkan Güney, Barbaros Hayrettin Ünlü, Hamit Ali, Ziya Ayhan, Canan Aslı Utine

**Affiliations:** 1 Department of Ophthalmology, Bakırköy Dr. Sadi Konuk Training and Research Hospital, Zuhuratbaba Neighborhood, Dr. Tevfik Sağlam Avenue No:11, Bakirköy, Istanbul, 34147 Turkey; 2 Department of Ophthalmology, Democracy University, İzmir, Turkey; 3 Izmir Health Technologies Development and Accelerator (BioIzmir), Dokuz Eylül University, İzmir, Turkey; 4 Department of Ophthalmology, Dr. Behçet Uz Children’s Training and Research Hospital, University of Health Sciences, İzmir, Turkey; 5 Department of Ophthalmology, Dünya Göz Hospital, İstanbul, Turkey; 6 Department of Ophthalmology, Menemen State Hospital, İzmir, Turkey; 7 Department of Ophthalmology, Dokuz Eylül University, İzmir, Turkey

**Keywords:** Cataract surgery, Surgically induced astigmatism, Surgeon seniority, Machine learning, Vector astigmatism

## Abstract

**Purpose:**

To evaluate whether surgeon seniority influences postoperative astigmatic change after phacoemulsification cataract surgery and to assess the performance of machine-learning models in predicting postoperative astigmatic outcomes using preoperative clinical data.

**Methods:**

This study included 169 eyes of 169 patients who underwent phacoemulsification cataract surgery. Surgeons were categorized as residents (*n* = 101) or specialists (*n* = 68). Astigmatism was quantified using both classical magnitude-based analysis and vector decomposition. Potential determinants of postoperative astigmatic change were evaluated using multivariable regression analyses. Machine-learning models, including Gradient Boosting, K-Nearest Neighbors, Decision Tree, and Support Vector Machine models, were used to predict classical and vector astigmatism outcomes. Model performance was assessed using mean absolute error, root mean square error, and the coefficient of determination.

**Results:**

Of the 169 eyes, 101 were operated on by residents and 68 by specialists. Postoperative astigmatic change did not differ significantly between resident- and specialist-performed procedures in either classical or vector analyses. Within the resident group, increasing surgical seniority was not associated with postoperative astigmatic change. In multivariable analysis, preoperative cylindrical error was the only significant predictor in the vector model, whereas no significant predictor was identified in the classical model. Overall, machine-learning models demonstrated limited predictive performance, with only modest utility for classical astigmatism and poor explanatory performance for vector outcomes.

**Conclusion:**

Surgeon seniority was not a significant determinant of surgically induced astigmatism after phacoemulsification cataract surgery. Machine-learning models based on preoperative clinical data provided only limited predictive value, particularly for vector astigmatism outcomes. Incorporating intraoperative variables may improve future predictive performance.

## Introduction

Modern cataract surgery is a refractive procedure, and even low residual astigmatism can measurably degrade uncorrected visual acuity and patient-reported visual quality. Evidence from large pseudophakic cohorts indicates that residual astigmatism of 0.50 D or more is clinically meaningful, reinforcing the need for precise astigmatism control [1]. The location and architecture of the incision are two biomechanical factors that affect surgically produced astigmatism. Prior studies have shown differences in astigmatic change between temporal, superior, and on-axis clear corneal approaches, and have identified baseline astigmatism and other host factors as contributors to postoperative astigmatic behavior [2–4]. Scalar magnitude by itself is not enough for a robust characterization of astigmatism because it is an axis-dependent phenomenon. Power-vector decomposition (J0/J45), as formalized by Thibos et al., enables orthogonal representation of cardinal and oblique components and provides a statistically tractable framework for direction-sensitive refractive outcomes [5, 6]. 

The contribution of surgeon experience to refractive outcomes after cataract surgery remains controversial. Although supervised training environments aim to ensure comparable surgical quality, classical literature suggests a learning-curve effect, particularly for operative efficiency and certain intraoperative parameters. However, large-scale real-world studies indicate that surgeon-related variability may be limited compared with patient-specific and preoperative factors in determining postoperative refractive outcomes. These findings raise uncertainty regarding the true impact of surgeon seniority on surgically induced astigmatism (SIA) [6–9]. 

In parallel, machine learning (ML) approaches have been explored for predicting refractive outcomes in cataract surgery, with inconsistent results. While some studies report modest improvements over traditional methods, others show limited or no added benefit, depending on dataset characteristics and model design. In particular, predicting axis-dependent astigmatic changes remains challenging, and the clinical utility of ML in this context is not yet well established [10]. 

This study aimed to evaluate whether surgeon seniority is associated with SIA after phacoemulsification and to assess the predictability of both classical and vector astigmatic outcomes using ML models based on preoperative variables.

## Methods

### Study design and population

This retrospective analytical study included 169 eyes from 169 patients who underwent cataract surgery at the Dokuz Eylul University Hospital, Department of Ophthalmology, between January 2020 and December 2025. All biometric measurements were obtained using the same optical biometry device (IOLMaster 500, Carl Zeiss Meditec, Jena, Germany). All cataract surgeries were performed with the Centurion Vision System (Alcon Laboratories, Fort Worth, TX, USA) using a standardized phacoemulsification technique based on the stop-chop method. Surgeons were categorized based on experience level into two groups: residents and specialists. All surgeons used an identical 2.4-mm clear corneal incision at the same predefined location for right and left eyes. The ophthalmology residency program has a minimum duration of 4 years, and residents begin performing phacoemulsification during their second year at our institution. Residents were classified as senior only if they were in at least their third year of residency and had previously performed at least 100 phacoemulsification procedures; residents who did not meet both criteria were classified as junior. The specialist surgeons had more than 20 years of independent cataract-surgery experience. All resident procedures were supervised by an attending ophthalmologist or a final-year senior resident but were completed entirely by the operating resident, including wound construction, without intervention or takeover. For residents, seniority (measured in months of training) was recorded to allow further evaluation of experience-related effects on measurements. Only one eye of each patient was included in the analysis, and demographic characteristics, including age and sex, were obtained from the institutional clinical database.

Patients were eligible for inclusion if they underwent uneventful cataract surgery with intraocular lens (IOL) implantation. Only cases with complete preoperative and postoperative biometric and refractive data were included in the analysis. In line with prior literature, only patients with satisfactory postoperative visual outcomes and reliable measurements were included, thereby minimizing potential bias from poor fixation or unreliable refraction.

Eyes were excluded if they had any ocular pathology other than cataract that could affect refractive outcomes or measurement accuracy, such as keratoconus, advanced glaucoma, diabetic retinopathy, or other retinal diseases, consistent with previously published methodologies. Cases with a history of previous ocular surgery, including refractive procedures, were excluded due to their known impact on corneal curvature and refractive predictability. Eyes with intraoperative or postoperative complications, or those requiring additional surgical interventions, were also excluded to ensure a homogeneous surgical cohort and to avoid confounding postoperative measurements. Advanced glaucoma was excluded because paracentral or central visual-field loss may be associated with unstable or eccentric fixation, potentially reducing the reliability of fixation-dependent refractive measurements [11]. Eyes with previous glaucoma filtration surgery were independently excluded under the prior-ocular-surgery criterion because trabeculectomy may alter axial length and keratometric astigmatism [12]. 

### Clinical variables and data extraction

All clinical and refractive parameters were extracted from the institutional database and compiled into a structured dataset. Preoperative measurements included spherical and cylindrical refractive errors, the astigmatism axis, keratometric values (K1 and K2), and vertical axis measurements. Postoperative parameters comprised the same set of measurements and were obtained at 1 month postoperatively, using a standardized protocol to ensure consistency. All measurements were performed with the same device (Nidek ARK 510-A autorefractometer, NIDEK Co., LTD., Japan) under uniform clinical conditions to minimize variability. Postoperative measurements were obtained at the 1-month visit, which was the standardized postoperative refractive assessment most consistently available in the institutional database. Postoperative refraction was performed as part of routine clinical care using a standardized protocol. Owing to the retrospective design of the study, the examiners were not specifically masked to the identity or seniority of the operating surgeon. The dataset included demographic and clinical variables, such as age, sex, eye laterality, surgeon category, and resident seniority, which were considered as potential covariates in the analysis.

Outcome measures were defined based on both scalar and vector representations of astigmatism. Classical keratometric astigmatism was defined as the absolute difference between the principal keratometric meridians (|K1 − K2|) for both preoperative and postoperative measurements. SIA was calculated as the difference between postoperative and preoperative keratometric astigmatism.

To account for axis-dependent changes in astigmatism, cylindrical power and axis values were transformed into orthogonal power vector components (J0 and J45). Vector astigmatism was defined as the change between preoperative and postoperative vectors, expressed as the differences in J0 and J45 components. Accordingly, analyses were performed using preoperative and postoperative J0/J45 values and their respective differences.

### Statistical analysis

Statistical analyses were performed using IBM SPSS Statistics for Windows, Version 25.0 (IBM Corp., Armonk, NY, USA). Because of the retrospective design, the sample size was determined by the number of consecutive eligible cases with complete data, and no a priori calculation or prospective randomization was performed. A sensitivity analysis based on 169 eyes (101 resident and 68 specialist cases), a two-sided α of 0.05, and 80% power showed that the study could detect a between-group effect size of Cohen’s d ≥ 0.44. The sample also exceeded the 150-eye threshold previously reported to provide stable performance in ML-based postoperative refraction prediction [13]. Continuous variables were expressed as mean ± standard deviation (SD), while categorical variables were presented as frequencies and percentages. Comparisons between residents and specialist groups were conducted using the independent samples t-test (Welch correction applied when appropriate). Categorical variables were analyzed using the chi-square test.

SIA was analyzed both as a scalar variable and as vector components (J0 and J45). Differences between preoperative and postoperative measurements were calculated and compared between groups. A two-sided p-value < 0.05 was considered statistically significant.

### Machine-learning models

A supervised ML framework was employed to predict both classical (scalar) and vector components of SIA using preoperative, demographic, and surgeon-related variables as predictors. The prespecified algorithms included Gradient Boosting (GB), Decision Tree (DT), K-Nearest Neighbors (KNN), and Support Vector Machine (SVM). The ML pipeline was implemented in Python (Python Software Foundation, Wilmington, DE, USA). Model performance was evaluated using mean absolute error (MAE), root mean square error (RMSE), and coefficient of determination (R²). Performance comparisons across models were conducted consistently for both scalar and vector outcomes, in alignment with the predefined endpoint structure used in the inferential statistical analysis.

## Results

A total of 169 eyes were included in this study (mean age: 69.71 ± 7.28 years). Sex distribution was balanced (82 women, 87 men), as was laterality (82 right, 87 left eyes). Surgeon groups comprised 101 resident-led and 68 specialist-led procedures. For the primary classical endpoint (keratometric induced astigmatism), resident and specialist groups showed comparable outcomes. Mean induced astigmatism was 0.25 ± 0.65 D in the resident group (complete-case *n* = 95) and 0.17 ± 0.70 D in the specialist group (*n* = 68), with no statistically significant difference (Mann–Whitney U, *p* = 0.488). When resident experience was analyzed as a continuous variable, seniority month was not associated with classical induced astigmatism (Spearman *r* = -0.04, *p* = 0.685), indicating no measurable learning-gradient effect for this endpoint within the observed training period.

Vectorial analysis similarly showed no between-group difference. Mean vector-induced astigmatism was 0.57 ± 0.39 D in resident cases and 0.55 ± 0.45 D in specialist cases (*p* = 0.387). In the resident subgroup, vector magnitude was not correlated with seniority month (*r* = 0.09, *p* = 0.36).

In multivariable modeling, preoperative cylindrical power emerged as the only statistically significant predictor in the vector framework (β = 0.12, SE = 0.03, t = 4.39, *p* < 0.001; 95% CI: 0.07 to 0.17), whereas age, sex, laterality, surgeon category, seniority month, and preoperative axis were not significant covariates (all *p* > 0.05; seniority month *p* = 0.09). In the classical keratometric model, no covariate reached significance (all *p* > 0.05). The results of the multivariable regression analyses for classical and vector-induced astigmatism are summarized in Table 1.

**Table 1:** Multivariable regression analysis of factors associated with classical and vector induced astigmatism

Variable	β	SE	t	*p* value	95% CI
**Classical induced astigmatism**					
Age	-0.01	0.01	-0.95	0.34	-0.03 to 0.01
Sex	-0.01	0.14	-0.06	0.95	-0.29 to 0.27
Eye laterality	-0.07	0.14	-0.50	0.62	-0.35 to 0.21
Surgeon category	0.62	0.42	1.49	0.14	-0.21 to 1.45
Seniority month	-0.01	0.02	-0.67	0.51	-0.05 to 0.03
Preoperative cylindrical error	0.02	0.05	0.45	0.65	-0.08 to 0.12
Preoperative axis	-0.00	0.00	-0.73	0.47	-0.00 to 0.00
**Vector induced astigmatism**					
Age	0.01	0.01	0.97	0.34	-0.01 to 0.02
Sex	-0.00	0.07	-0.02	0.98	-0.15 to 0.15
Eye laterality	-0.02	0.07	-0.33	0.74	-0.17 to 0.12
Surgeon category	0.03	0.22	0.15	0.88	-0.41 to 0.47
Seniority month	0.02	0.02	0.12	0.09	-0.04 to 0.12
Preoperative cylindrical error	0.12	0.03	4.39	**< 0.001**	0.07 to 0.17
Preoperative axis	-0.00	0.00	-1.50	0.14	-0.00 to 0.00

For ML prediction of classical induced astigmatism, Gradient Boosting achieved the best performance (MAE: 0.41, RMSE: 0.51, R²: 0.06). For vector-induced astigmatism, Gradient Boosting also yielded the lowest error (MAE: 0.27, RMSE: 0.33, R²: -0.51). Despite lower absolute errors in the best-performing models, R² values indicated limited explained variance, particularly for vector outcomes, suggesting that preoperative variables alone captured only a small proportion of postoperative variability. The performance of the ML models for predicting classical and vector-induced astigmatism is presented in Table 2.

**Table 2:** Performance of machine-learning models for prediction of classical and vector induced astigmatism

Model	MAE	RMSE	*R*²
**Classical induced astigmatism**			
Gradient Boosting	0.405	0.508	0.055
Decision Tree	0.443	0.560	-0.124
SVM	0.554	0.678	-0.626
K-Nearest Neighbors	0.573	0.704	-0.738
**Vector induced astigmatism**			
Gradient Boosting	0.267	0.330	-0.510
Decision Tree	0.293	0.356	-0.637
SVM	0.391	0.482	-1.514
K-Nearest Neighbors	0.364	0.465	-1.377

MAE, mean absolute error; RMSE, root mean square error; R², coefficient of determination; SVM, support vector machine

Feature-importance analysis supported this pattern: in classical modeling, preoperative cylindrical error, preoperative axis, K1 and K2 values, and seniority duration ranked among the most informative variables; in vector modeling, preoperative steep axis was the dominant contributor. Collectively, these findings indicate that surgeon seniority does not materially influence the magnitude of induced astigmatism. At the same time, preoperative cylinder remains the principal measurable signal, and ML models provide only partial predictive utility. Table 3 presents the relative feature-importance rankings derived from the Gradient Boosting and Decision Tree models for predicting classical and vector-induced astigmatism.

**Table 3:** Feature importance rankings of tree-based machine-learning models for classical and vector induced astigmatism prediction

Variable	Gradient Boosting	Decision Tree
**Classical induced astigmatism**		
Preoperative cylindrical error	0.467	0.384
Preoperative steep axis	0.242	0.346
Preoperative K2	0.168	0.074
Seniority month	0.051	0.094
Preoperative spherical error	0.112	0.095
Preoperative K1	0.093	0.051
Age	0.076	0.082
Eye laterality	0.015	0.003
Sex	0.010	0.006
Surgeon category	0.001	0.002
**Vector induced astigmatism**		
Preoperative steep axis	0.474	0.479
Preoperative cylindrical error	0.164	0.211
Preoperative K2	0.077	0.163
Age	0.068	0.057
Preoperative K1	0.063	0.035
Preoperative spherical error	0.032	0.059
Seniority month	0.022	0.026
Eye laterality	0.017	0.005
Sex	0.012	0.007
Surgeon category	0.002	0.029

## Discussion

The principal finding of this study is that surgeon seniority was not significantly associated with the magnitude of SIA after phacoemulsification. The absence of significant differences between resident and specialist groups in both classical and vector outcomes, together with the lack of correlation between resident month and vector astigmatism, suggests that seniority alone is not a dominant determinant of postoperative astigmatic change. This is consistent with large real-world datasets showing that surgeon-level effects on refractive outcomes are relatively small compared with patient-level factors [6, 8]. Therefore, rather than contradicting the established cataract surgery learning-curve literature [7, 9], our findings suggest that experience-related differences may be more readily reflected in operative efficiency, case duration, or intraoperative performance than in postoperative SIA itself. On the other hand, the lack of a significant difference between residents and specialists may partly reflect selection effects. Since only uneventful surgeries with reliable postoperative measurements were analyzed, complicated cases and eyes with less predictable refractive outcomes were excluded. Such exclusions may have occurred more frequently in the resident group, while more complex eyes may also have been preferentially operated on by specialists. As a result, the analyzed cohort may not fully reflect the true distribution of surgical difficulty, potentially masking differences related to surgeon seniority.

The absolute difference in mean classical induced astigmatism between resident- and specialist-performed procedures was 0.08 D (0.25 D vs. 0.17 D). Residual astigmatism of approximately 0.50 D or greater may measurably affect uncorrected visual outcomes [1], and a randomized comparison suggested that toric IOL correction may become beneficial from approximately 0.75 D of preoperative corneal astigmatism [14]. These thresholds relate to residual or preoperative astigmatism rather than directly to SIA and therefore should not be treated as interchangeable; nevertheless, the observed 0.08 D between-group difference is small in relation to them and is unlikely, by itself, to alter routine toric-IOL candidacy or refractive planning. Individual outliers may still be clinically relevant despite the small group-level difference.

Even within the resident subgroup, seniority month was not associated with either classical induced astigmatism or vector astigmatic change, and it did not remain significant in multivariable analysis. This suggests that a linear month-by-month learning-curve effect could not be demonstrated for SIA within the observed training period. However, this should not be taken to mean that there is no learning curve in cataract surgery. Rather, it may indicate that SIA is too narrow a refractive endpoint to capture such a curve, particularly in a standardized and supervised surgical setting. This interpretation is consistent with the resident cataract surgery literature, where experience-related gains are often more evident in broader procedural outcomes, such as operative time, efficiency, and intraoperative performance [7–9]. A possible explanation is that seniority month may be too crude a proxy for the microsurgical skills most relevant to SIA, including incision geometry, wound construction, and tissue handling [15]. Accordingly, our findings are better interpreted as showing no detectable linear learning gradient for SIA, rather than no surgical learning curve overall.

Preoperative cylindrical power was the only variable that remained significant in multivariable analysis, and only within the vector model. This suggests that postoperative astigmatic behavior is shaped primarily by the baseline corneal–refractive state rather than by routine surgeon- or patient-level descriptors. The fact that the signal emerged specifically in the vector framework is clinically meaningful because vector-based analysis preserves axis information and may better capture the baseline astigmatic pattern; consistent with this, prior regression-based work has shown that preoperative vector components contribute to predicting postoperative astigmatism [16, 17]. Prior studies have likewise shown that both incision-related effects and postoperative astigmatic outcomes are conditioned by the amount and pattern of baseline astigmatism [4, 18, 19]. By contrast, the absence of independent effects for age, sex, laterality, surgeon category, and seniority indicates that these commonly recorded variables have limited standalone explanatory value for postoperative astigmatic variation under standardized surgical conditions. Taken together with large-scale evidence suggesting relatively small surgeon-level effects on refractive outcomes [6], our findings support the view that the preoperative corneal profile may be a more important measurable determinant of postoperative astigmatic behavior than surgeon designation alone in this setting.

A further strength of the present study is that astigmatism was evaluated not only as a scalar magnitude but also through J0/J45 power-vector decomposition. This distinction is clinically and methodologically important because scalar analysis reduces astigmatism to magnitude alone, whereas vector analysis preserves directional information and allows orthogonal assessment of cardinal and oblique components [17]. As a result, eyes with similar scalar astigmatism may still differ meaningfully in terms of meridional redistribution, a feature that can be obscured when only magnitude-based outcomes are considered [5, 17]. In our cohort, however, surgeon seniority was not associated with postoperative astigmatic change in either analytical framework. The consistency of this negative result across both scalar and vector approaches strengthens the interpretation that the absence of a seniority effect is unlikely to be merely a consequence of endpoint definition. At the same time, the vector-based findings provide additional biological plausibility for the role of baseline corneal configuration: preoperative cylindrical power remained the only significant determinant in the multivariable vector model, and axis-related information became more prominent in vector-oriented predictive analysis, underscoring the inherently axis-dependent nature of astigmatism. This interpretation is broadly consistent with contemporary vector-analysis literature, which supports direction-sensitive characterization of astigmatic change and highlights the substantial interindividual variability and limited predictability of SIA after modern cataract surgery [5, 20]. Taken together, these findings suggest that incorporating both classical and vector astigmatism yields a more comprehensive assessment of postoperative corneal behavior and that the lack of association with surgeon seniority is methodologically robust rather than analysis-specific.

For classical astigmatism prediction, Gradient Boosting performed best among the tested models, yet its clinical and explanatory value remained limited. Despite acceptable MAE and RMSE values, the R² of 0.06 indicates that only a very small fraction of postoperative variability was captured. Thus, low error did not necessarily translate into high explanatory value. This pattern suggests that the model detected weak average-level structure in the data, but not enough stable signal to support strong individualized prediction. A broadly similar caution has been reported in cataract ML studies, where small reductions in absolute prediction error do not necessarily translate into statistically or clinically superior overall performance [10]. Given that the present framework relied on preoperative variables alone, these findings may also suggest a ceiling effect for preoperative-only prediction of postoperative astigmatic change, particularly because explainable ML analyses of cataract prediction error have shown that conventional biometric variables often account for only a limited proportion of residual variance [21]. The limited sample size may also have reduced the model’s ability to learn subtle nonlinear relationships. In addition, given the modest sample size, model evaluation is inherently more vulnerable to overfitting and to instability in out-of-sample performance estimates; the negative R² observed for the vector outcome indicates that the model performed worse than a simple mean-based prediction on held-out data, and should be interpreted as a signal of limited generalizability given the available sample and feature set rather than evidence of a coding or validation error. Larger, ideally multicenter, datasets with more robust cross-validation would allow more stable estimates of model performance for both classical and vector SIA outcomes. Notably, preoperative cylinder, surgeon category, and seniority duration ranked among the most informative variables, indicating that these factors remain partially relevant; however, their contribution appears insufficient to generate clinically strong predictions on their own.

Another notable finding was the particularly weak performance in predicting vector astigmatism. Although Gradient Boosting achieved the lowest absolute error for the vector outcome, the negative R² indicates that the model failed to explain meaningful postoperative variance and, in practical terms, performed worse than a naive mean-based prediction. This finding is clinically plausible rather than unexpected. Unlike scalar astigmatism, vector astigmatism is inherently axis-dependent, and small axis-related changes can produce marked shifts in J0/J45 components; measurement variability in astigmatism may further reduce model stability [17, 22]. In addition, the present ML framework relied exclusively on preoperative, demographic, and surgeon-related variables in a relatively small sample. In contrast, vector astigmatic change after cataract surgery is likely influenced by intraoperative wound-related factors that were not captured in the dataset, including incision meridian and location, tunnel configuration, and other aspects of wound architecture; stromal hydration may also contribute indirectly through its effects on incision morphology [19, 23, 24]. From this perspective, the model was asked to predict a direction-sensitive and surgically modulated postoperative outcome without access to several of its most plausible determinants. The fact that axis orientation emerged as the dominant feature in vector modeling further supports this interpretation: the model appears to recognize that direction matters, but lacks sufficient information to predict how surgery ultimately alters that directional pattern. Therefore, the poor vector-model performance should not be interpreted merely as a technical failure of machine learning, but rather as evidence that vector astigmatism is a more complex endpoint whose prediction likely requires richer intraoperative and possibly early postoperative data beyond preoperative inputs alone [21].

An additional important finding concerns the interpretation of feature-importance patterns derived from the ML models. In the classical model, preoperative cylinder, surgeon category, and seniority duration ranked among the most informative variables, whereas axis orientation was the dominant contributor in vector prediction. However, these rankings should not be equated with strong or independent clinical determinants. Given the low explanatory performance of the models—particularly the very weak R² values—feature-importance results are better interpreted as limited, model-specific predictive signal rather than as evidence of robust causal or independently predictive effects [25–27]. This distinction is especially relevant in the present study, because variable importance reflects model reliance within a specific algorithmic setting and may be influenced by correlated predictors or weak shared patterns that do not necessarily emerge as significant in conventional inferential models [25, 26]. Within this framework, the prominence of axis orientation in vector prediction is biologically and optically expected, since power-vector decomposition (J0/J45) is inherently axis-dependent and directly incorporates directional astigmatic information [17]. By contrast, the appearance of surgeon-related variables among the leading contributors in the classical model, despite their lack of significance in regression analysis, suggests at most a limited and context-dependent contribution. In practical terms, these variables may carry weak interaction-based or shared predictive signals that are not sufficiently strong to serve as stable, independent determinants of postoperative astigmatic change. Taken together, the feature-importance findings support a cautious interpretation: baseline corneal/refractive structure may provide the main measurable signal, whereas surgeon-related variables may exert only small, context-sensitive effects that become visible in prediction models but do not consistently withstand inferential testing. In settings with limited predictive performance, the reliability and stability of feature-importance rankings may also be constrained, further supporting cautious interpretation of these patterns [27].

From a clinical standpoint, the present findings suggest that, under standardized phacoemulsification conditions, surgeon seniority alone is unlikely to be a major determinant of SIA. The lack of significant differences between resident- and specialist-performed procedures, together with the absence of a measurable association between resident seniority month and postoperative astigmatic change, indicates that refractive optimization should not be framed primarily around surgeon title or stage of training. Instead, a more actionable strategy likely lies in patient-specific planning and technical execution, particularly regarding the preoperative corneal/refractive profile and incision-related wound characteristics. In this context, the emergence of preoperative cylindrical power as the only significant predictor in multivariable analysis reinforces the view that postoperative astigmatic behavior is more strongly related to baseline astigmatic configuration than to routine surgeon-level descriptors. Likewise, the modest performance of classical ML models and the poor explanatory capacity of vector prediction further suggest that postoperative astigmatism is unlikely to be adequately explained by seniority-based variables alone.

The study has several important strengths. It directly compared resident and specialist surgeons within the same standardized surgical environment, while also evaluating resident experience as a continuous variable rather than relying solely on categorical grouping. Astigmatism was assessed using both scalar and vector frameworks, allowing direction-sensitive behavior to be examined beyond magnitude alone. In addition, the combination of conventional inferential statistics and ML approaches provided complementary perspectives: the former identified the absence of a robust seniority effect and the relative importance of preoperative cylinder, whereas the latter suggested that some variables may retain a partial predictive signal despite limited overall explanatory power. This multimodal analytical design strengthens the study’s internal coherence and makes the negative findings more informative than merely descriptive.

Several limitations should also be acknowledged. The retrospective design, single-center setting, and relatively limited sample size may have reduced statistical power and limited model generalizability. In addition, the cohort was intentionally restricted to uncomplicated cases with complete and reliable measurements, which improved internal uniformity but may also have introduced a selection effect and reduced applicability to more complex real-world cataract surgery. Another important limitation is the absence of detailed intraoperative variables, such as exact incision meridian, tunnel length, use of stromal hydration, phaco energy, and wound architecture, all of which are biologically plausible determinants of postoperative astigmatic change and may be particularly relevant for vector outcomes. This is a particularly consequential omission, since incision meridian and tunnel architecture are widely regarded as principal biomechanical determinants of SIA and bear directly on the primary aim of this study; several of the strongest previously reported predictors of induced astigmatism, including incision length, exact incision location, wound hydration, phaco energy, and cumulative dissipated energy, are intraoperative rather than preoperative parameters [19, 20]. Because none of these variables were captured in the present retrospective dataset, the limited predictive performance of the machine-learning models is an expected consequence of the available preoperative feature set rather than an unanticipated shortcoming of the modeling approach itself, and should be interpreted accordingly. In addition, corneal thickness and biomechanical properties were not included in the analysis, although these factors may also influence corneal response to surgery and the magnitude of SIA. A further limitation relates to the timing of postoperative assessment: measurements were obtained at 1 month after surgery, and although most wound-healing-related corneal change after a standardized clear corneal incision is thought to occur within the first postoperative weeks, some degree of further corneal remodeling may continue beyond this point. A longer follow-up (e.g., ≥ 6 weeks to 3 months) could have provided additional confirmation of the stability of the reported SIA values; however, because the same 1-month time point was applied uniformly to both resident- and specialist-performed procedures, this is unlikely to have introduced a differential bias between groups. Because the analysis included one eye per patient, inter-eye dependence was not a major methodological concern in the present dataset.

**In conclusion**, based on our findings, surgeon seniority was not a significant determinant of surgically induced astigmatism after standardized phacoemulsification. Postoperative astigmatic change was primarily associated with baseline corneal characteristics, with preoperative cylindrical error emerging as the only consistent predictor in vector analysis. The limited performance of machine-learning models further suggests that preoperative variables alone are insufficient to explain postoperative variability, particularly for axis-dependent outcomes. These findings support a shift toward patient- and technique-centered factors rather than surgeon seniority in understanding SIA.

Future studies should adopt larger, preferably multicenter, prospective designs and integrate richer intraoperative parameters into both statistical and ML models. Such an approach may improve SIA prediction and provide a more clinically useful framework for refractive precision after cataract surgery.

## Data Availability

The data presented in this study are available upon request from the corresponding author (D.O.) due to privacy considerations.

## Abbreviations

CI: Confidence interval
DT: Decision Tree
GB: Gradient Boosting
IOL: Intraocular lens
J0 and J45: Power-vector components of astigmatism
KNN: K-Nearest Neighbors
MAE: Mean absolute error
ML: Machine learning
RMSE: Root mean square error
R²: Coefficient of determination
SD: Standard deviation
SIA: Surgically induced astigmatism
SVM: Support Vector Machine
